# Impact of Silver Diamine Fluoride Therapy on Oral Health-related Quality of Life of Uncooperative Preschool Children: A Prospective Study

**DOI:** 10.3290/j.ohpd.b927709

**Published:** 2021-01-28

**Authors:** Apathsakayan Renugalakshmi, Thilla Sekar Vinothkumar, Fatimah Balqasim Hakami, Ruba Mohammed Salem, Amnah Ali Qadri, Zahra Mohammed Harbosh, Zaki Hakami

**Affiliations:** a Assistant Professor, Division of Pedodontics, Department of Preventive Dental Sciences, College of Dentistry, Jazan University, Jazan, Saudi Arabia. Idea, study design, analysed the data, wrote, read and approved the final manuscript.; b Assistant Professor, Department of Restorative Dental Sciences, College of Dentistry, Jazan University, Jazan, Saudi Arabia. Study design, analysed the data, contributed substantially to discussion, wrote, read and approved the final manuscript.; c Intern, College of Dentistry, Jazan University, Jazan, Saudi Arabia. Collected and analysed the data, read and approved the final manuscript.; d Associate Professor, Department Head of Preventive Dental Sciences, College of Dentistry, Jazan University, Jazan, Saudi Arabia. Study design, supervision, organization, contributed substantially to discussion, read and approved the final manuscript.

**Keywords:** children, dental caries, mothers, silver diamine fluoride, quality of life

## Abstract

**Purpose:**

Preschool children with early childhood caries (ECC) frequently exhibit extreme dental anxiety and fear, posing a considerable challenge to paediatric dentists for their treatment. The aim of this study was to evaluate the influence of silver diamine fluoride (SDF) treatment on the oral health-related quality of life (OHRQoL) of uncooperative preschool children using an Arabic version of the Early Childhood Oral Health Impact Scale (A-ECOHIS).

**Materials and Methods:**

A pre-validated A-ECOHIS was used to assess the sensitivity and responsiveness. Fifty-one children, uncooperative with conventional dental care, underwent SDF treatment; their mothers answered the A-ECOHIS before and 4 weeks after treatment. Based on the global transition rating (GTR), the mothers rated their child’s oral health condition following SDF treatment.

**Results:**

SDF effectively arrested caries after 4 weeks in all children. There was a statistically significant reduction in mean scores of the total A-ECOHIS, child impact scale and family impact scores at follow-up (Wilcoxon signed-rank test; p ˂ 0.001). There were statistically significant changes in the mean GTR of children’s oral health.

**Conclusions:**

A-ECOHIS was sensitive and responsive to SDF treatment. SDF statistically significantly improved the OHRQoL of uncooperative preschool children.

Early childhood caries (ECC) is a detrimental disease involving young children which burdens societies worldwide.^26^ Its prevalence has recently shown a widespread increase in children aged 2–5 years, making them a target group for the Federation Dentaire Internationale.^15^ Generally, the prevalence of caries has been reported to be 73% in Saudi Arabia.^4^ A recent study conducted in Jazan, a small city located in the southern part of Saudi Arabia, has revealed an alarming caries prevalence among children (females: 93.9%; males: 89.4%).^32^

Impediments to caries management include behavioural issues (anxiety and fear) due to young age, low cooperation, or the family’s limited access to care and financial restrictions.^1,27^ Although state-of-the-art behavioural management procedures, including sedation and general anesthesia are promising, they are associated with unforeseen complications (affecting brain development, relatively high fatality), difficult logistics and limited access.^17,22,29^

Over the past decade, silver diamine fluoride (SDF) has been frequently discussed in the literature due to its simple application protocol with a minimum number of visits and potential anti-cariogenic properties in primary dentition.^16,20^ In addition to the cariostatic activity, the distinguishing feature of SDF is that it also simultaneously prevents the formation of new caries when compared with other materials.^28^ SDF has an inherent disadvantage of staining the carious lesions following its application.^7^ Hence, it is imperative to inform the children and their parents about the unpleasant aesthetics.^20^ It has been stated that the black staining of carious teeth is a serious problem resulting in the social isolation of children.^5^ In a recent study conducted in Saudi Arabia, parents judged staining on the anterior teeth to be aesthetically inacceptable (90%) and refused this treatment option (66-92%).^2^

Negligence of caries treatment is often correlated with poor oral health-related quality of life (OHRQoL).^31^ The Early Childhood Oral Health Impact Scale (ECOHIS) is an OHRQoL assessment tool introduced especially for young children directed towards their parents.^24^ The Arabic version of this tool (A-ECOHIS) had been validated^14^ and recommended for in vivo studies to ascertain the effect of various interventions on OHRQoL.^13^

Sensitivity refers to ability of the assessment tool to identify a significant change in outcome following a particular intervention based on changes in the distribution of scores.^3^ Responsiveness refers to competency of the assessment tool to change in relation to global transition rating (GTR) of changes in oral health. The GTR acts as a tool of measuring the patient’s self-observed health condition over a particular time period.^19^ A-ECOHIS has demonstrated significant sensitivity and responsiveness to dental rehabilitation under general anesthesia.^13^ It may be speculated that general anesthesia has a positive influence on A-ECOHIS, especially on the child’s comfort. Hence, situations demand revalidation of A-ECOHIS for preschool-aged uncooperative children in a dental clinic setting in terms of SDF treatment. Moreover, to the authors’ best knowledge, there is no information regarding the effect of SDF treatment on OHRQoL in Arabic pre-school children.

Therefore, the purpose of this study was to evaluate the impact of SDF treatment on OHRQoL of uncooperative children using A-ECOHIS. Moreover, we evaluated sensitivity and responsiveness of A-ECOHIS to SDF treatment. The null hypothesis tested was that there is no difference in the OHRQoL of uncooperative children suffering from ECC before and after SDF intervention.

## Materials and Methods

Ethical approval was obtained for this prospective study from the institutional review board (REC41/1-013). The study was performed in accordance with the ethical standards of the 1964 Declaration of Helsinki and its later amendments or with comparable ethical standards.

### Participants

This study was conducted in the dental clinic of the College of Dentistry, Jazan University, from December 2019 to February 2020. A total of 51 uncooperative children (Frankl rating scale for dental anxiety and fear score 2 = negative behaviour^6^) between 24 and 72 months of age who reported for primary dental care were selected for the study using convenience sampling. These children were resistant to conventional operative care, requiring management otherwise under general anaesthesia.

As a part of inclusion criteria, children had to be medically fit and not have any known sensitivity to any components of SDF, present at least one carious lesion based on the International Caries Detection and Assessment System (ICDAS) with scores ranging from 1 to 5. Using ICDAS, carious lesions in the primary dentition were categorised as initial carious lesions (ICDAS 1 or 2); noncavitated lesions (ICDAS 3 or 4); or active (soft) cavitated carious lesions extending into the dentin (ICDAS 5).^18^ Exclusion criteria included: children who are highly resistant to conventional operative care (Frankl rating 1 = definitely negative behaviour) requiring management under general anaesthesia; those who are cooperative for operative care (Frankl rating 3 = positive and rating 4 = definitely positive); with symptomatic pulpitis or tooth mobility; genetic dental defects such as amelogenesis imperfecta or dentinogenesis imperfecta; previous exposure to SDF treatment; or unable to attend the recall visits after 4 weeks. Informed consent was sought and obtained from the mothers prior to participation in the study.

### Clinical Examination

Three investigation teams, each consisting of an investigator and an assigned recorder, conducted the intraoral examination and documented the caries exposure according to ICDAS. The investigators were trained and calibrated by performing duplicate examinations on 10% of subjects pertaining to caries exposure and lesion status for the affected teeth (inter-examiner Kappa = 0.92).

### Measures

Initially, A-ECOHIS was completed independently by mothers, without aid from the dental team, to provide details on the children’s demographic characteristics and OHRQoL. Mothers were again requested to respond after four weeks. The questionnaire consists of 13 components divided into two sections, namely, the child impact section (CIS) and parent impact section (PIS). CIS has 9 components divided into four descriptive domains [child symptom (CSy) – 1; child function (CF) – 4; child psychology (CP) – 2; child self-image/social interaction (CS) – 2] and PIS has 4 components split into two domains [parent distress (PD) – 2 and family function (FF) – 2]. The feedback options for A-ECOHIS were as follows: 0 = never; 1 = hardly ever; 2 = occasionally; 3 = often; 4 = very often; 5 = not sure. The total A-ECOHIS scores and individual domain scores were calculated. Questionnaires with a score of 5 either more than twice in CIS or once in FIS were disqualified from the study.^24^ The total A-ECOHIS, CIS, and FIS scores can vary from 0-52, 0-36, and 0-16 respectively. The lower the A-ECOHIS score, the weaker is the influence of oral health on OHRQoL.

The children were recalled after 4 weeks to evaluate the efficacy of SDF. Asymptomatic teeth that had become black and hard with no change in ICDAS score were considered positive outcomes. In addition to A-ECOHIS, mothers were asked to respond the GTR through the question ‘How has your child’s oral health changed after undergoing SDF treatment?’ on a five-point Likert scale with the options ‘much worse’, ‘a little worse’, ‘same’, ‘a little improved’ and ‘much improved’.

### SDF Treatment

All children recruited for the study were treated with SDF (Fagamin, Tedequim SRL; Cordoba, Argentina) in the dental clinic setting. After careful cleaning and drying of carious tooth surfaces, the gingiva adjacent to the tooth was coated with petroleum jelly to prevent discolouration. A drop of solution was painted on with a microbrush and left undisturbed for 1 min. Systemic absorption was prevented by wiping the extra material using cotton rolls. The child was instructed not to eat or drink for half an hour post treatment. Improvement in a child’s behaviour during SDF application was recorded and verified based on the Frankl rating scale. Information on immediate postoperative adverse effects, such as difficulty in swallowing, nausea, gingival irritation or burns within 24–48 h, was collected by the recorder via telephone.

### Data Analysis

Statistical analyses were performed using the IBM SPSS Statistics version 23.0 software (SPSS; Chicago, IL, USA) at a significance level of p ˂ 0.05. The sensitivity of A-ECOHIS was estimated by detecting the variation in distribution of scores using Wilcoxon’s signed-rank test. Effect size (ES) was calculated to reveal the strength of the statistical change.^10^ The similarity in variance pattern between A-ECOHIS and GTR perceived by the mothers was verified to determine the responsiveness of A-ECOHIS. The minimally important difference (MID) was determined as the difference in mean values of mothers who reported ‘same’ in the GTR before and after SDF treatment for each A-ECOHIS domain, in order to substantiate the results. The MID values were rounded to the nearest whole number and the proportion of children with mean values of each domain greater than the corresponding MID values were expressed in percent.

## Results

### Patient and Family Characteristics

Table 1 presents the demographic characteristics of children and their mothers. Of the 51 children selected, 20 (39%) were males and 31 (61%) were females. The mean age and standard deviation (SD) of children and their mothers were 5.2 (1.09) and 34.7 (6.17) years, respectively. Most of the children in our study were between 5 and 6 years (62.7%). Forty-six (90.2%) children displayed an improved Frankl rating from 2 to 3 during the SDF treatment. The remaining 5 (9.8%) children still exhibited a Frankl rating pf 2.159 teeth (3.12 ± 0.33 teeth per child) and received SDF treatment. The initial ICDAS code for majority of the teeth was 5 (43.4%) and all the teeth (100%) that underwent SDF treatment showed positive outcomes, where the carious tooth surfaces became hard and black.

**Table 1:** Demographic characteristics of 51 children and their mothers

Characteristics		N or Mean (SD or %)
Gender	Males	20 (39%)
	Females	31 (61%)
Children’s age (years)	Males	5. 5 (0.90)
	Females	5.1 (1.18)
	< 3 years	6 (11.8%)
	3 to 4	7 (13.7%)
	4 to 5	6 (11.8%)
	5 to 6	32 (62.7%)
Mother’s age (years)	≤ 30	20 (39.2%)
	≤ 40	26 (51 %)
	≤ 50	5 (9.8%)
ICDAS Code	2	12 (7.5%)
	3	33 (20.8%)
	4	45 (28.3%)
	5	69 (43.4%)
Teeth	Anterior	119 (74.8%)
	Posterior	40 (25.2%)
Frankl rating	2	5 (9.8%)
	3	46 (90.2%)

### Effect of SDF Treatment on Children OHRQoL

Table 2 presents sensitivity of A-ECOHIS to SDF before and after treatment. There was a statistically significant reduction (p ˂ 0.001) in the total (64.3%), child impact section (64.3%) and family impact section (24.9%) scores following treatment. The effect size for all domains was large, except for the family impact section and family function (0.50 and 0.71), which was moderate. The largest effect size was observed in parental distress (1.55), followed by child symptoms (1.39).

**Table 2:** Sensitivity of A-ECOHIS to pre- and post-SDF treatment

ECOHIS domains	Range	Pre-SDF Mean (SD)	Post-SDF Mean (SD)	p-value#	Observed effect mean (SD)	Effect size^a^
Total ECOHIS	0–52	22.53 (10.21)	8 (9.40 )	<0.001**	14.53 (13.79)	1.42
Child impact section	0–36	13.94 ( 7.07)	4.98 ( 5.86)	<0.001**	8.96 (9.08)	1.27
Child symptoms	0–4	2.59 (1.13)	1.02 (1.09)	<0.001**	1.57 (1.42)	1.39
Child function	0–16	6.74 (3.18)	2.49 (2.74)	<0.001**	4.25 (3.71)	1.34
Child psychology	0–4	3.16 (2.33)	0.90 (1.57)	<0.001**	2.25 (2.91)	0.97
Child self-image	0–4	1.45 (2.11)	0.57 (1.40)	0.018*	0.88 (2.51)	1.31
Family impact section	0–16	8.59 (4.24)	3.02 (4.05)	<0.001**	5.57 (5.44)	0.50
Parental distress	0–4	5.63 (2.47)	1.80 (2.29)	<0.001**	3.82 (3.31)	1.55
Family function	0–4	2.96 (2.45)	1.22 (2.11)	<0.001**	1.74 (2.66)	0.71

#p-value obtained through Wilcoxon signed-rank test. *p < 0.05, **p < 0.001. ^a^Effect size as described by Cohen’s d, which is interpreted as small (0.20–0.50), medium (0.50–0.70), and large (> 0.70).

Responsiveness of A-ECOHIS was evaluated by comparing the GTR given by mothers with the A-ECOHIS scores (Table 3). The five-point scale of GTR was integrated and presented under three broad categories as ‘improved’, ‘same’ and ‘worse’. Forty-two (82.3%) mothers perceived their children’s condition as ‘improved’ following treatment, 8 (15.7%) reported ‘same’ and 1 (2%) perceived the condition as ‘worse’. Among the mothers in the ‘improved’ category, there was a very highly statistically significant change in the scores of A-ECOHIs in all domains (p ˂ 0.001). There was no statistically significant difference for A-ECOHIS total (p = 0.057) for the mothers of ‘same’ category. There was a gradual decline in the effect sizes from the ‘improved’ category to ‘same’.

**Table 3:** Responsiveness of A-ECOHIS compared to GTR following SDF treatment

ECOHIS domains	Range	Pre-SDF Mean (SD)	Post-SDF Mean (SD)	p-value#	Observed Effect Mean (SD)	Effect size^a^
IMPROVED (N = 42)						
Total ECOHIS	0-52	18.31 (9.99)	7.29 (7.30)	< 0.001**	11.02 (12.09)	1.10
**Child impact section**	0-36	13.26 (7.13)	4.79 (4.60)	< 0.001**	7.48 (7.03)	1.05
Child symptoms	0-4	2.57 (1.19)	0.79 (1.01)	< 0.001**	1.79 (1.42)	1.50
Child function	0-16	5.29 (1.40)	3.10 (0.81)	< 0.001**	2.19 (0.96)	1.56
Child psychology	0-8	2.69 (1.07)	0.62 (0.78)	< 0.001**	2.07 (1.54)	1.93
Child self-image	0-8	1.71 (1.28)	0.29 (0.52)	< 0.001**	1.43 (1.36)	1.12
**Family impact section**	0-16	6.05 (3.67)	2.50 (3.22)	< 0.001**	3.55 (4.64)	0.97
Parental distress	0-8	3.14 (0.74)	1.79 (0.94)	< 0.001**	1.36 (1.44)	1.83
Family function	0-8	2.90 (1.30)	0.71 (0.88)	< 0.001**	2.19 (1.41)	1.68
SAME (N=8)						
Total ECOHIS	0-52	18.03 (9.18)	13.9 (9.48)	0.057	4.13 (8.09)	0.45
**Child impact section**	0-36	12.13 (6.26)	9.13 (5.67)	0.001*	3.00 (1.22)	0.48
Child symptoms	0-4	2.75 (0.71)	2.25 (1.28)	0.275	0.50 (1.20)	0.71
Child function	0-16	6.50 (4.40)	5.10 (4.11)	0.009*	1.40 (1.26)	0.32
Child psychology	0-8	1.75 (0.81)	1.13 (0.51)	0.019*	0.62 (0.48)	0.77
Child self-image	0-8	1.13 (0.89)	0.63 (0.60)	0.164	0.50 (0.68)	0.56
**Family impact section**	0-16	5.88 (2.50)	4.75 (3.51)	0.083	1.13 (2.50)	0.45
Parental distress	0-8	2.88 (1.01)	3.13 (1.41)	0.633	-0.25 (1.02)	-0.25
Family function	0-8	2.38 (1.06)	1.63 (0.98)	0.029*	0.75 (0.73)	0.70
WORSENED (N=1)						

# p-value obtained through paired t-test, *p < 0.05, **p < 0.001. ^a^Effect size as described by Cohen’s d, which is interpreted as small (0.20–0.50), medium (0.50–0.70), and large (>0.70).

Table 4 presents the minimally important difference values of A-ECOHIS for children determined with ‘same’ category as the minimum reference using both the anchor-based (GTR) and distribution-based approach (observed mean difference). Based on the findings, the minimally important difference value was 4 for the total A-ECOHIS score, 3 for the CIS section and 1 for the remaining domains.

**Table 4:** Distribution of children based on Minimally Important Difference (MID)

A-ECOHIS	MID	N (%)
Total ECOHIS	4	41 (80.4)
**Child impact section**	3	38 (74.5)
Child symptoms	1	36 (70.6)
Child function	1	37 (72.5)
Child psychology	1	36 (70.6)
Child self-image	1	20 (39.2)
**Family impact section**	1	42 (82.4)
Parental distress	1	42 (82.4)
Family function	1	34 (66.7)

## Discussion

The present short-term prospective clinical study displayed the cariostatic efficacy of SDF in the primary teeth of uncooperative children aged 2 to 6 years. Various factors responsible for uncooperative behaviour of children in the dental office are the noise from rotary and hand instruments, phobia toward dental injections and parents’ anxiety.^28^ Mothers rather than fathers were selected for responding to the questionnaire due to their better understanding of their children’s oral health.^25^

The various proposed mechanisms of action for SDF are reduction in the growth of cariogenic bacteria, interference with dentin collagen degradation, decrease in demineralisation and promotion of remineralisation in both enamel and dentin.^33^ Furthermore, an increase in subsurface fluoride concentration has been observed following SDF application.^28^ All teeth that were exposed to SDF became coal-black in appearance with carious lesions arrested, irrespective of the ICDAS code at the end of 4 weeks, which is consistent with the previous studies.^9,30^

SDF treatment has been recommended for young, treatment-resistant preschool children, and most parents accepted the blackish discolouration due to SDF as preferable to advanced management strategies such as sedation or general anesthesia.^11^ In our study, there were no incidents of adverse effects following SDF treatment.

Sufficient reviews have endorsed favorable results for SDF in caries control and prevention in preschool children.^16,23^ Most of the studies reporting the efficacy of SDF have excluded children who were uncooperative (negative behaviour), except for a recent short-term clinical study in which the behaviour rating was not followed to categorise the children.^9^ In our study, children with a Frankl rating of 2 were specifically selected to appreciate the magnitude of SDF impact on the OHRQoL of uncooperative children. Interestingly, 46 children (90.2%) showed behavioural improvement to Frankl rating 3 during the procedure. The combination of clear explanation and simplicity of the treatment technique resulted in successful management of such uncooperative children. Moreover a ‘no caries removal’ approach was followed in order to reduce the chairside time for children and hence also their dental anxiety.^8^ Nevertheless, dental treatment under general anesthesia remains the preferred treatment by paediatric dentists for children with Frankl rating 1 and 2 whose carious teeth are painful and infected.^13^

In the present study, pre-treatment A-ECOHIS scores were high, which reflects the alarming prevalence of ECC and poor OHRQoL of young children in Saudi Arabia, which is in agreement with the study by Farsi et al.^13^ However, the pre-treatment total ECOHIS, CIS and FIS scores for children in our study were higher than that found in previous studies in Hong Kong^12^ and Brazil.^30^ The post-treatment total scores of our study revealed the effectiveness of SDF treatment (ES: 1.42), which is consistent with that of Vollu et al^30^ (ES: 0.58), in contrast to the negative outcome discussed by Duangthip et al^30^ (ES: -0.08). All the domains of the A-ECOHIS were equally responsive to SDF treatment, thereby proving its sensitivity. Accordingly, OHRQoL improved after SDF intervention, leading to a rejection of the null hypothesis. The magnitude of change as measured by ES was large in all of the domains, except in child’s CS and FIS domains, which exhibited moderate change. This closely resembles the results of Vollu et al.^30^ This could be attributed to the satisfaction of children and their parents due to alleviation of dentin sensitivity, in spite of black staining.

Interestingly, 82.3% of mothers responded that their child’s oral health had ‘improved’ after SDF, which contradicts the results of a Chinese-ECOHIS (39.8%).^12^ This can be explained by the difference in sampling population between kindergarten children (Chinese-ECOHIS) and the needy children coming to dental clinics with direct dentist-parent interaction. The mean change in total A-ECOHIS, CIS scores, FIS scores for the ‘improved’ category ranged from 2.5 to 3.5 times that of ‘same’ category, suggesting the adaptability of A-ECOHIS with GTR. Furthermore, the total A-ECOHIS scores for parents who reported the ‘same’ GTR was not statistically significantly different, thereby confirming the responsiveness of A-ECOHIS as a valid tool for evaluating the OHRQoL following SDF intervention. Hence, A-ECOHIS scores of all domains for parents who reported the ‘same’ category were taken as the minimum reference for estimating the MID. The MID values allow clinicians to assess the strength of a treatment result. The MID value for total A-ECOHIS, CIS and FIS following SDF treatment represented a 4-, 3- and 1-point change, respectively, which can act as a reference point for clinicians and researchers pursuing further investigations.

The strength of the present study was the participation of 100% of the patients (no dropouts), despite dealing with young and uncooperative children. A potential limitation is the short-term follow-up of patients for four weeks. However, promising caries arrest results of SDF have also been obtained in a short-term clinical trial conducted by Milgrom et al.^21^

## Conclusion

The A-ECOHIS is a useful tool for evaluating the OHRQoL of uncooperative Arabic children, and exhibited sensitivity and responsiveness to SDF intervention. SDF treatment showed a positive outcome on the carious lesions and eventually on the OHRQoL, thereby recommending it as a potential treatment modality. Moreover, SDF intervention is a promising method to nudge uncooperative children towards conventional restorative treatment. Further investigations are required to monitor the behaviour of these uncooperative children during conventional restorative treatment and subsequently the impact of SDF on the durability of restorations.
